# Automated Protocols for Macromolecular Crystallization at the MRC Laboratory of Molecular Biology

**DOI:** 10.3791/55790

**Published:** 2018-01-24

**Authors:** Fabrice Gorrec, Jan Löwe

**Affiliations:** ^1^Laboratory of Molecular Biology, Medical Research Council

**Keywords:** Biochemistry, Issue 131, X-ray crystallography, protein crystallization, high-throughput screening, fully automated system, nanoliter droplets, vapor diffusion, initial screen, nanoliter dispenser, liquid handler, crystal optimization, 4-corner method, additive screening

## Abstract

When high quality crystals are obtained that diffract X-rays, the crystal structure may be solved at near atomic resolution. The conditions to crystallize proteins, DNAs, RNAs, and their complexes can however not be predicted. Employing a broad variety of conditions is a way to increase the yield of quality diffraction crystals. Two fully automated systems have been developed at the MRC Laboratory of Molecular Biology (Cambridge, England, MRC-LMB) that facilitate crystallization screening against 1,920 initial conditions by vapor diffusion in nanoliter droplets. Semi-automated protocols have also been developed to optimize conditions by changing the concentrations of reagents, the pH, or by introducing additives that potentially enhance properties of the resulting crystals. All the corresponding protocols will be described in detail and briefly discussed. Taken together, they enable convenient and highly efficient macromolecular crystallization in a multi-user facility, while giving the users control over key parameters of their experiments.

**Figure:**
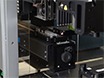


## Introduction

X-ray crystallography is extensively applied to further advance our understanding of biological and disease mechanisms at the atomic level and to subsequently assist rational approaches to drug discovery^1^. For this, purified and concentrated (2-50 mg/mL) macromolecular samples of protein, DNA, RNA, other ligands, and their complexes are trialed for their propensity to form ordered three-dimensional lattices through crystallization^2^,^3^,^4^. When high quality crystals are obtained that diffract X-rays, the crystal structure may be solved at near atomic resolution^5^,^6^. Crucially, the conditions to crystallize a novel sample cannot be predicted and the yield of high quality crystals is usually very low. An underlying reason is that many samples of interest have challenging biochemical properties, which make them unstable on the corresponding timescale for crystallization (typically a few days). Finally, the process is compounded by the time required to produce samples and sample variants, and to optimize their purification and crystallization^7^,^8^.

A crystallization condition is a solution with a precipitant that reduces sample solubility, and conditions often also contain buffers and additives. Hundreds of such reagents are well suited to alter the parameters of the crystallization experiments as they have low propensity to interfere with sample integrity (such as protein or nucleic acid unfolding). While testing millions of combinations of crystallization reagents is not feasible, testing several to many screening kits - formulated with various strategies^9^,^10^ - is possible with miniaturized trials and automated protocols. In this perspective, the most amenable technique is probably vapor diffusion with 100-200 nL droplets sitting on a small well above a reservoir containing the crystallization condition (25-250 µL), implemented in specialized crystallization plates^11^,^12^. The protein sample and condition are often combined in a 1:1 ratio for a total volume of 200 nL when setting up the droplets in the upper-wells. Robotic nanoliter protein crystallization can be implemented with alternative techniques and plates such as the under-oil batch^13^ and the Lipidic Cubic Phase^14^ (the latest one being applied specifically to trans-membrane proteins that are very poorly soluble in water).

The crystallization facility at the MRC-LMB was started in the early 2000s and an early summary of our automated protocols was presented in 2005^15^. A historical introduction to protein crystallization was presented and also an outline of the advantages of robotic nanoliter approach (then a novel approach to routine experimentation). Since macromolecular crystallization is essentially a stochastic process with very little or no useful prior information, employing a broad variety of (suitable) initial conditions increase the yield of quality diffraction crystals^16^. Besides, an often-overlooked advantage of a large initial screen is to significantly reduce the need for optimization of samples and crystals in many cases. Of course, one may still need to proceed with optimization of some initial conditions later. Typically, the concentration of reagents and the pH are then systematically investigated. More reagents can also be introduced into the optimized condition(s) to further alter parameters of crystallization. Certainly, one should attempt crystallization with a sample freshly prepared, hence the corresponding protocols must be straightforward and available any time.

Here, two fully automated systems designed at the MRC-LMB (systems 1 and 2) and the corresponding protocols are fully described. The main application of these two systems is initial screening by vapor diffusion in sitting drop crystallization plates. System 1 integrates a liquid handler, an automated carousel to stock plates, an inkjet printer for plate labeling and an adhesive plate sealer. On the system 1, 72 96-well plates are filled with commercially available screening kits (80 µL of condition transferred to reservoir from a starting volume of 10 mL in test tubes), labeled and sealed. The plates are then stored in a 10 °C incubator where they are available to users anytime (as initial screens called 'LMB plates').

System 2 integrates a liquid handler, a nanoliter dispenser, and an adhesive plate sealer. On the system 2, sitting droplets (100-1,000 nL) for vapor diffusion experiments are produced by combining conditions and the sample in the upper-wells of 20 48- or 96-well plates pre-filled with conditions. This means 1,920 initial screening conditions are trialed when using 20 LMB plates on the system 2.

Robots are also used individually for the optimization of selected conditions, and the corresponding semi-automated protocols are also described. The 4-corner method^17^ is employed routinely to produce optimization screens. The corresponding protocol first requires the manual preparation of 4 solutions ('A, B, C, and D'). Two linear gradients of concentrations (for two main crystallization agents) are then automatically generated directly into the reservoirs of a crystallization plate. For this, a syringe-based liquid handler dispenses the 4 corner solutions at different ratios.

To further optimize a condition, one can employ additive screens that potentially enhance the properties of resulting crystals^18^. Two approaches are available for additive screening: a protocol starting with additives dispensed into the reservoirs of crystallization plates before setting up the droplets (protocol 1)****and another protocol where the additive screen is dispensed directly onto the droplets (protocol 2).

Other useful developments that were initiated at the MRC-LMB to facilitate automated macromolecular crystallization, are also presented. Essentially, crystallization plates and associated devices such as a stackable Society of Biomolecular Screening (SBS) lid that minimizes evaporation of conditions when using the system 2.

For brevity, it is assumed that users are familiar with the basic functions and maintenance of the nanoliter dispenser, the inkjet printer, and the adhesive plate sealer. Unless stated otherwise, plates on the deck of the robots are positioned such that the well A1 ('A1-corner') is towards the back-left corner of a plate carrier.

## Protocol

### 1. The two fully automated systems (initial screening)

1. **System 1: preparation of 72 96-well crystallization plates filled with screening kits (LMB plates)**
  1. Prior to performing the procedure, ensure that the system's robots are initialized and their controlling software is open. Turn on the chiller of the tube-cooling carrier about 30 minutes before the main program will be started.
  2. Place a test plate on the motorized SBS carrier of the plate sealer. Run the plate sealer and check that the film is properly applied. Repeat this test twice more to verify that the plate sealer is ready.
  3. Then, add 20 L of deionized water to the main container of the liquid handler. Disconnect the coupling inserts from the small container (20% ethanol rinsing solution) and connect the inserts to the main container.
  4. Enter the appropriate screen name (**Table 1**) on the inkjet printer touchscreen. Then, open and close the carousel door to trigger the carousel rotation. Open the door when the first stack presents itself.
  5. Fully load the first stack with 22 crystallization plates, each with column 1 facing out. Fully load the next two stacks in the same way.
  6. Load the remaining 6 plates in the highest positions of the fourth stack. Then, close the carousel door.
  7. Verify that the chiller display indicates 14 °C. Gently and repeatedly invert the selected screening kits for 1 minute. Then, open the front panel of the liquid handler.
  8. Open the tubes and place them in the cooling carrier according to the standard 96-well layout (A1, A2, *etc*.). Upon placing each tube in the carrier, place its lid on a tray in the same 96-well layout.
  9. Cross-check the tube positions and ensure that all tubes are level and settled in the carrier. Then, close the front panel.
  10. In the 'Startup' window of the liquid handler software, select 'Run maintenance' and open the flushing program. Run the program to flush the 20% ethanol from the system and to wash the outsides of the liquid-dispensing tips.
  11. When flushing is complete, click 'Cancel' to return to 'Startup'. Select 'Run an existing process' and click 'Start your selection'. Open the 'MRC kit dispensing' program. Fill in '18' for 'Instances' in the configuration screen and run the program.
  12. Monitor the system as the first four plates are labeled, filled, and sealed.
  13. Once all 72 plates have been prepared and placed back in the carousel, switch the coupling inserts from the main container to the small container. Run the 'Start up flush' program (see step 1.1.10).
  14. Turn off the chiller and discard the empty screening kit tubes. Carefully remove the 72 prepared plates from the carousel. Discard incorrectly-filled or poorly-sealed plates. Store the ready-to-use plates at 10 °C.
2. **System 2: setting up crystallization droplets (100 nL protein + 100 nL condition) from a single sample in 20 LMB plates**
  1. First, ensure that the system's robots are on, the nanoliter dispenser is initialized with its software open, and the liquid handler's method manager is open. Turn on the microtube-cooling carrier about 15 minutes before the main program will be started.
  2. Place a test plate on the motorized SBS carrier of the plate sealer. Run the plate sealer and verify that the plate is properly sealed. Test the plate sealer three times.
  3. Then, run the nanoliter dispenser to set 100-nL droplets of test solution in a test plate. Check under a microscope that the droplets are set correctly. Close the nanoliter dispenser software and remove the strip-holder block from the deck.
  4. Next, insert in the custom-designed plate holder (see **Representative Results**: Crystallization Devices Developed at the LMB) the LMB plate with the highest relative quantity of volatile reagents in its conditions (**Table 1**). Remove the adhesive film from the plate.
  5. Open the liquid handler front panel and place the unsealed plate on the deck, at the back of the first sliding carrier (Sliding carriers may be pulled out for ease of access).
  6. Cover the plate with an SBS aluminum lid. Settle the lid towards the rear left corner of the carrier by applying gentle pressure to the opposite corner of the lid.
  7. Unseal, load into the sliding carriers, and cover the remaining 19 plates in the same way, working from most to least volatile conditions. Once the microtube-cooling carrier is at 4 °C, as indicated by a green light, remove its cover.
  8. Cut off the lid of a microtube containing (at least) 440 µL of protein sample (**Table 2**). Ensure that the sample has no foam above the meniscus, as this will interfere with the liquid detection system. Place the tube in Position 1 of the microtube-cooling carrier.
  9. Place a PCR plate on the liquid handler deck in front of the plate-moving adapter carrier. Then, close the front panel.
  10. After loading the deck, ensure that the nanoliter dispenser deck is clear of the strip-holder block and that the carriers at the back of the 50-µL tip stacks are clear of the aluminum SBS lids.
  11. In the liquid handler 'Method Management' interface, select 'Setup plates'. Monitor the initialization of both systems and fill in the run parameters. Follow the guidelines of the Method Management interface (prompts, figures and a tip-management system help with preparation).
  12. Double-check that all required components are ready, and then start the process.
  13. Monitor the system as the nanoliter dispenser sets the drops in the first plate and the plate sealer subsequently seals the plate.
  14. Once all 20 plates have been prepared with crystallization droplets and automatically returned to the sliding carriers, open the front panel and gently remove the plates. Check that the plates are correctly sealed before storing them for crystallization.
  15. Clean the SBS lids with a 20% ethanol solution before stacking them on the left-hand side of the liquid handler for storage. Discard the PCR plate and the microtube.
  16. Turn off the microtube-cooling carrier and wipe away the condensation. Leave a paper towel on top of the cooler surface to absorb further condensation. Then, replace the carrier cover and close the front panel.

### 2. Optimization of the conditions

1. **Syringe-based liquid handler: producing two linear gradients of concentrations into the reservoirs of a crystallization plate (the 4-corner method).**
  1. First, ensure that the liquid handler is on and initialized with its software open.
  2. On the 'GRADIENT' tab: Open the required program, select the crystallization plate type and the final volume in the reservoirs (**Table 3**). *The advanced setting for 'max shot vol' should be lowered from 6,000 to 3,000 when using solutions containing [isopropanol] > 10% v/v and [MPD] > 20% v/v.*
  3. Prepare the syringes. Place a piston in each syringe (pointed ends down) and insert the back of the syringes into the designated grooves underneath the robot head. Twist a syringe clockwise to lock it in position (The program will start only with all the required syringes attached correctly).
  4. Prepare the troughs. Remove the stainless-steel frame and insert the troughs. The 4 positions on the left correspond to the 4 corners A, B, C, D. Switch to 'SET UP' tab which displays the volumes of solutions required in each syringe (on **Table 3**, 0.5 mL dead volume was added to the volumes displayed). Pour the corner solutions into their respective troughs and place the frame back on the deck (the frame holds in position with 2 small magnets located at the front of the deck). *An alternative way to proceed with this step is to pour the solutions into the troughs when they are already placed on the deck.*
  5. Place the crystallization plate on the motorized SBS carrier.
  6. Click 'ASPIRATE' and wait for this step to be completed (when pistons stopped on their way up).
  7. Switch to the 'RUN' tab and run program.
  8. Upon completion of the program, go back to the SET UP tab and click 'REMOVE': the system purges the syringes from leftover solutions, and then lifts the pistons all the way up. *'PURGE' may be requested instead of 'REMOVE'; this will leave the pistons at the bottom position, ready to aspirate more solutions with the same syringes.*
  9. Remove the syringes by twisting them anticlockwise.
  10. Discard syringes and troughs in the appropriate bin (or rinse them with deionized water and then 20% v/v ethanol solution for reuse).
  11. Seal the plate and place it onto the microplate mixer for 3 min at 1,000 rpm, or 10 min when highly viscous solutions are being mixed. The plate is ready for setting up crystallization droplets on the nanoliter dispenser.
2. **Additive screening protocol 1 (setting up crystallization droplets in a 96-well crystallization plate pre-filled with additive screen)**
  1. First, prepare the condition with initial concentrations of reagents increased by 10% (min. vol. 15 mL when transferring the condition from a container onto the additive screen with the liquid handler).
  2. Ensure that the liquid handler is ready to operate. Open the program 'Add screen to additives' for a single plate (enter volume in reservoirs: '72 µL'). *Prompts, figures and a tip-management system (12 x 1,000 µL tips required) help with making sure the robot is ready to operate according to the selections.*
  3. Retrieve the additive screen from the -20 °C incubator and remove its aluminum seal immediately (use the plate holder), then place the plate on the deck of the liquid handler. *The program operates according to a portrait layout (the plate is placed with the A1-corner located in the front-left of the carrier).* Also place also the container filled with the condition. Run the program.
  4. Once the reservoirs of the plate have been filled, place it onto the microplate shaker and run the program. Rinse the container of condition with deionized water and 20 % ethanol for reuse.
  5. **Set up the droplets on the nanoliter dispenser**. First, unseal the crystallization plate (use the plate holder). Then, place the plate and the 8-well protein strip in the first position of the strip-holder block. *The 'Setup' tab on the controlling software displays the actual positions of each component on the deck*. Load each well of the strip with protein sample according to the drop size required (**Table 4**). *Run the program to prepare droplets.*
  6. Upon completion of the program, remove the plate from the deck and seal it immediately (use the plate sealer, 3-inch wide adhesive tape). Discard the strip in the appropriate bin.
  7. Assess the size, shape, and centering of the droplets under the microscope before storage.
3. **Additive screening protocol 2 (setting up crystallization droplets in a 96-well crystallization plate with a re-usable additive screen)**
  1. First, prepare the additive screen: leave the corresponding frozen 96-well cell culture plate to thaw at room temperature for 40 min. Then, centrifuge the additive screen at 1,000 x g for 2 min.
  2. Prepare the condition (min. vol. 15 mL when transferring the condition from a container onto the additive screen with the liquid handler).
  3. Fill the reservoirs of a 96-well crystallization plate with the condition. On the liquid-handler, proceed the same way as protocol 1, step 2.2.2, but enter '80 µL' for the volume in reservoirs.
  4. Set up the droplets on the nanoliter dispenser with 3 components on the deck (the plate containing the additive screen, along with the crystallization plate and the 8-well protein strip in the first position of the strip-holder block, **Table 4**).
  5. Seal the cell culture plate containing the screen with an aluminum sheet and place it back in the -20 °C incubator.
  6. Assess the size, shape, and centering of the droplets under the microscope before storage.

## Representative Results

**1. System 1 and LMB plates**

Figure 1A shows the system 1 based on a liquid handler operating with a liquid-system (deionised water). The liquid-system comprises a container, a pump, tubing, 8 syringes equipped with valves and 8 fixed tips. The liquid class settings were optimized to aspirate/dispense a broad variety of solutions and multi-dispense into 4 plates (multi-dispense requires a relatively large excess of aspirated volume). A 22 L water container and a smaller container (5 L) with 20 % v/v ethanol are stored underneath the system to feed the liquid-system. Each container is equipped with two coupling inserts. One insert (colored blue) feeds the system with liquid, the other one (red) is a flow-back to reduce excess pressure. After use, flushing with water and then 20% v/v ethanol solution prevents microbial growth. A cooling unit (also located underneath the system) is connected to a custom-built tube-cooling carrier. System 1 is 2050 mm wide, including the carousel, 760 mm deep and 88 mm high. An additional 550 mm is required in the front for the worktable that holds the control unit of the inkjet printer and the adhesive plate sealer. Additional space is also needed next to the system for the controlling PC. The program to produce 72 pre-filled plates (*i.e.* 18 rounds of 4 plates) takes 3 h and 50 min.

Figure 1B is a close-up of the main deck that is equipped with 8 fixed tips (Figure 1C) mounted on an automated pipetting arm, a second arm with a gripper, a tip wash station, 2 x 4-position carriers for SBS plates and the tube-cooling carrier. The main program processes 4 plates at a time that are taken out automatically from the carousel and placed on the deck where they are filled with crystallization conditions (80 µL in reservoirs, Figure 1D). Liquid levels are automatically detected as the tips are conductive. While the liquid handler dispenses conditions into a set of plates, another set of empty plates is taken out from the carousel, labeled and placed onto the main deck. A small custom-built holder and window in the rear panel of the liquid handler were required to position the printer head and its sensor at the back of the main deck. The 8 tips are flushed and washed with water from the main container after each dispensing step that consists of 4 aliquots in the corresponding columns of reservoirs. After 4 plates have been filled, they are automatically sealed and placed back in their original position in the carousel. The plate sealer is triggered by a specific driver (called by the controlling software). The sealer uses a roll of 3-inch wide adhesive tape which is applied to a plate with rollers under mechanical pressure. Upon completion of the program, the pre-filled plates are removed manually from the carousel stacks and stored in a 10 °C incubator located within the facility.

**Figure:**
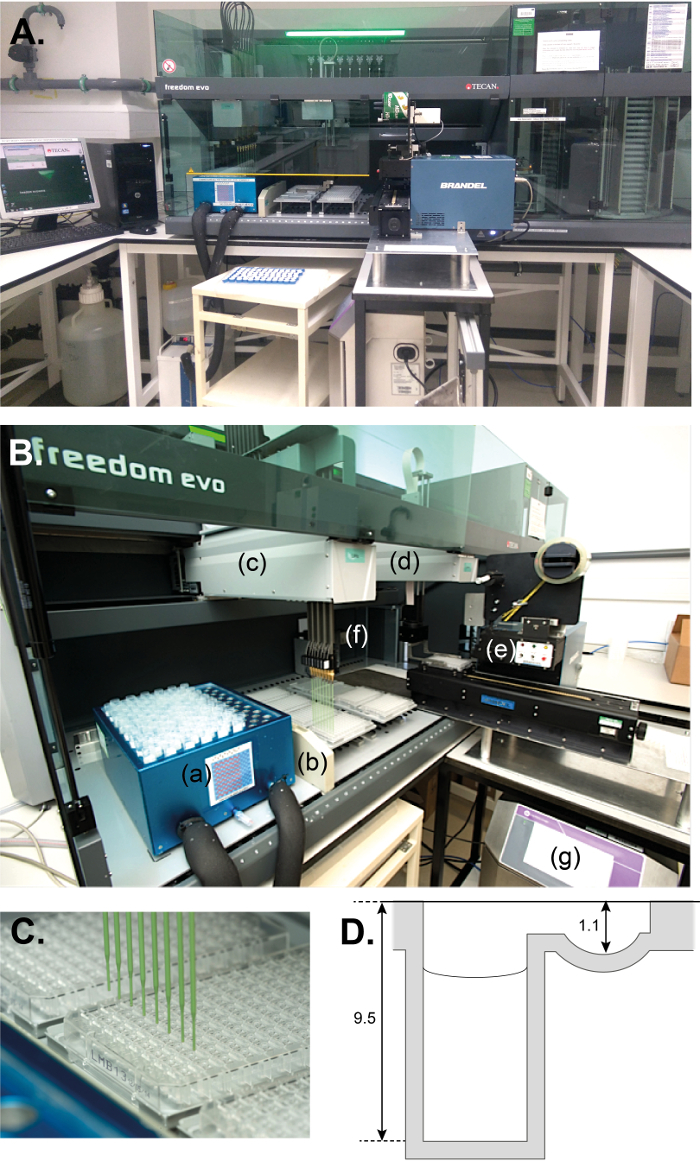

Figure 1**: Filling the reservoirs with initial screening kits.** (**A**) Overview of the fully automated system 1. The carousel is a separate automated unit with plate stacks and it is housed in a well made of clear acrylic sheets on the right-hand side the main deck. (**B**) The main deck of the liquid handler in operation with (**a**) the tube-cooling carrier (connected to a chilling unit underneath, not shown), (**b**) the fast wash station (connected to drainage, not shown), (**c**) the pipetting arm filling 8 reservoirs of a 96-well crystallization plate, (**d**) the gripper arm bringing a filled plate onto (**e**) the motorized SBS carrier of the plate sealer. In the back of the deck is (**f**) the printing head of the inkjet printer connected to (**g**) the inkjet control unit underneath the sealer. (**C**) A labeled plate (name of the screen and date of production) being filled with crystallization conditions by 8 conductive fixed tips made of Polytetrafluoroethylene-coated stainless steel. (**D**) Cross-section of a sealed crystallization well. The reservoir contains 80 µL of crystallization condition (while the upper well is empty). Reservoir and upper-well depth specifications are in millimeters. Please click here to view a larger version of this figure.

**Table 1** shows the different formulations for the commercially available screening kits in test tubes. The kits are used to fill the reservoirs of 96-well crystallization plates (LMB01-LMB22) on a regular basis with the system 1.

**Table:** 

**Plate name**	**kit name**	**supplier**	**catalog number**	**tubes**	**basic description**
LMB01	Crystal Screen 1	Hampton Research	HR2-110	48	Sparse Matrix (pH 4.6-8.5)
	Crystal Screen 2	Hampton Research	HR2-112	48	Stochastic sampling (pH 4.6-9.0)
LMB02	Wizard 1	Rigaku	1009530	48	Stochastic sampling (pH 4.5-10.5)
	Wizard 2	Rigaku	1009531	48	Stochastic sampling (pH 4.5-10.5)
LMB03	Grid Screen Ammonium Sulfate	Hampton Research	HR2-211	24	Grid screen, [AmS] = 0.8-3.2 M and buffers pH 4.0-9.0
	Grid Screen PEG/LiCl	Hampton Research	HR2-217	24	Grid screen, [PEG 6000] = 0-30 %w/v, conc. LiCl = 1.0 M and buffers pH 4.0-9.0
	Quick Screen	Hampton Research	HR2-221	24	Grid screen, [NaKPO4] = 0.8-1.8 M at pH 5.0-8.2
	Grid Screen Sodium Chloride	Hampton Research	HR2-219	24	Grid screen, [NaCl] = 1.0-4.0 M and buffers pH 4.0-9.0
LMB04	Grid Screen PEG 6000	Hampton Research	HR2-213	24	Grid screen, [PEG 6000] = 5-30 %w/v and buffers pH 4.0-9.0
	Grid Screen MPD	Hampton Research	HR2-215	24	Grid screen [MPD] = 10-65 %w/v and buffers pH 4.0-9.0
	MemFac	Hampton Research	HR2-114	48	Sparse Matrix for membrane proteins (pH 4.6-8.5)
LMB05	PEG-Ion	Hampton Research	HR2-126	48	Grid screen, [PEG 3350] = 20 %w/v and various salts at 0.2 M (no buffers)
	Natrix	Hampton Research	HR2-116	48	Incomplete factorial (pH 5.6-8.5)
LMB06	Crystal Screen Lite	Hampton Research	HR2-128	48	Crystal Screen 1 with one-half of the original precipitant concentrations
	Custom Lite screen	Molecular Dimensions	n/a	48	Additional conditions with low precipitant concentrations
LMB07	Wizard Cryo 1	Rigaku	1009536	48	Stochastic sampling with conditions cryoprotected using low MW PEGs (pH 4.5-9.4)
	Wizard Cryo 2	Rigaku	1009537	48	Stochastic sampling with conditions cryoprotected using low MW PEGs (pH 4.5-10.1)
LMB08	JBS1	JenaBioScience	CS-101L	24	Incomplete factorial based on various PEGs (pH 4.6-9.0)
	JBS2	JenaBioScience	CS-102L	24	Incomplete factorial based on PEG 4000 (pH 4.6-8.5)
	JBS3	JenaBioScience	CS-103L	24	Incomplete factorial based on PEG 4000 (pH 4.6-8.5)
	JBS4	JenaBioScience	CS-104L	24	Incomplete factorial based on medium MW PEGs (pH 6.5-8.5)
LMB09	JBS5	JenaBioScience	CS-105L	24	Incomplete factorial based on heavy MW PEGs (pH 6.5-9.5)
	JBS6	JenaBioScience	CS-106L	24	Incomplete factorial based on AmS (pH 4.6-8.5)
	JBS7	JenaBioScience	CS-107L	24	Incomplete factorial based on MPD (pH 4.6-8.5)
	JBS8	JenaBioScience	CS-108L	24	Incomplete factorial based on MPD and ethanol (pH 4.6-8.5)
LMB10	JBS9	JenaBioScience	CS-109L	24	Incomplete factorial based on common salts and 2-propanol (pH 4.6-8.5)
	JBS10	JenaBioScience	CS-110L	24	Incomplete factorial based on common salts (pH 4.6-8.5)
	Clear Strategy Screen 1 pH 4.5	Molecular Dimensions	MD1-16LMB	24	Grid screen with various PEGs
	Clear Strategy Screen 1 pH 5.5	Molecular Dimensions	MD1-16LMB	24	Grid screen with various PEGs
LMB11	Clear Strategy Screen 1 pH 6.5	Molecular Dimensions	MD1-16LMB	24	Grid screen with various PEGs
	Clear Strategy Screen 1 pH 7.5	Molecular Dimensions	MD1-16LMB	24	Grid screen with various PEGs
	Clear Strategy Screen 1 pH 8.5	Molecular Dimensions	MD1-16LMB	24	Grid screen with various PEGs
	Clear Strategy Screen 2 pH 4.5	Molecular Dimensions	MD1-16LMB	24	Grid screen with various PEGs
LMB12	Clear Strategy Screen 2 pH 5.5	Molecular Dimensions	MD1-16LMB	24	Grid screen with various PEGs
	Clear Strategy Screen 2 pH 6.5	Molecular Dimensions	MD1-16LMB	24	Grid screen with various PEGs
	Clear Strategy Screen 2 pH 7.5	Molecular Dimensions	MD1-16LMB	24	Grid screen with various PEGs
	Clear Strategy Screen 2 pH 8.5	Molecular Dimensions	MD1-16LMB	24	Grid screen with various PEGs
LMB13	Index	Hampton Research	HR2-144	96	Small sparse matrix and grid screens (pH 3.0-9.0)
LMB14	SaltRX 1	Hampton Research	HR2-107	48	Grid screen including 22 unique salts versus salt concentration and pH (4.1-9.0)
	SaltRX 2	Hampton Research	HR2-109	48	Grid screen including 22 unique salts versus salt concentration and pH (4.1-9.0)
LMB15	MemStart	Molecular Dimensions	MD1-21	48	Sparse matrix for membrane proteins (pH 4.0-10.0)
	MemSys	Molecular Dimensions	MD1-25	48	Grid screen for membrane proteins (mostly PEGs, pH 3.5-9.5)
LMB16	JCSG+	Qiagen	130720	96	Sparse Matrix (pH 4.0-10.0)
LMB17	MORPHEUS screen	Molecular Dimensions	MD1-46	96	Grid screen including mixes of additives and cryoprotected conditions (pH 6.5-8.5)
LMB18	Pi minimal screen	JenaBioScience	CS-127	96	Incomplete factorial (pH 4.0-9.5)
LMB19	Pi-PEG screen	JenaBioScience	CS-128	96	Incomplete factorial for membrane proteins (pH 4.8-8.8)
LMB20	MORPHEUS II screen	Molecular Dimensions	MD1-91	96	Grid screen including mixes of additives (heavy atoms) and cryoprotected conditions (pH 6.5-8.5)
LMB21	LMB crystallization screen	Molecular Dimensions	MD1-98	96	Sparse matrix including conditions selected from LMB publications
LMB22	MORPHEUS III screen	Molecular Dimensions	n/a	96	Grid screen including mixes of additives (drug compounds) and cryoprotected conditions (pH 6.5-8.5)

**Table 1: Formulations of the kits found into LMB plates**. Each commercial screening kit consists of 24/48/96 conditions initially in test tubes. The LMB plates are stocked in 10 °C incubators located within the facility where they are available to users anytime.

**2. System 2 and requirements for setting up droplets**

Figure 2A shows the system 2 based on a liquid handler^19^ operating with positive displacement and disposable tips. The disposable tips are supplied in stackable racks suitable for automated handling. A 3-position deck nanoliter dispenser^20^ was integrated on the right-hand side of the system. Aspirate/dispense on the nanoliter dispenser also operates with positive displacement using disposable microsyringes (supplied in large spools). As a stand-alone robot, a removable strip-holder block is used to load protein sample(s). For the fully automated process, a 384-well PCR plate replaces the strip-holder. A similar adhesive plate sealer to system 1 was integrated on the left-hand side. The sealer and nanoliter dispenser stand on custom-built, raised worktables in order for the main gripper to reach the plate carriers of these two integrated robots (a window had to be cut in both side panels of the liquid handler for the gripper to gain access outside the main deck). The main program calls specific drivers to trigger nanoliter dispensing programs and the sealer in due time. The system 2 is 2,850 mm wide, 800 mm deep and 800 mm high. Additional space is needed next to the robot for the display of the controlling PC. Two waste bins are located underneath the system for the tips and microsyringes discarded during the process (the controlling PC is also stored underneath). An important characteristic of the system 2 is the overall layout, which retains easy access to the three robots so that they can be used individually for the optimization protocols described earlier, or other protocols described elsewhere such as crystallization of membrane proteins in lipidic mesophases^21^ and random microseed matrix screening^22^.

Figure 2B is a close-up of the deck that is equipped with a single robotic arm. The arm integrates 12 independent pipetting probes and the main gripper. The positions of the probes can be controlled individually in one direction in order to access different locations along the axis between the probes or even single tubes. The probes can pick up either disposable tips (1-12) or a pair of plate-moving adaptors. Two sets of 4 stacks containing 50 µL disposable tips are initially loaded. Liquid levels are automatically detected as the tips are conductive. The plate-moving adaptors are designed with 2 sharp pins on the inside that form an optional gripper when needed. On the main deck is located a 24-position microtube cooling carrier for the sample, although only 1-2 positions are used here. In addition, there is a carrier for the PCR plate and also 2 storage carriers for stackable, custom-built SBS lids (Figure 2C). Finally, there are 4 sliding carriers, each with 5 locations for crystallization plates (4 x 5 = 20 plates).

**Figure:**
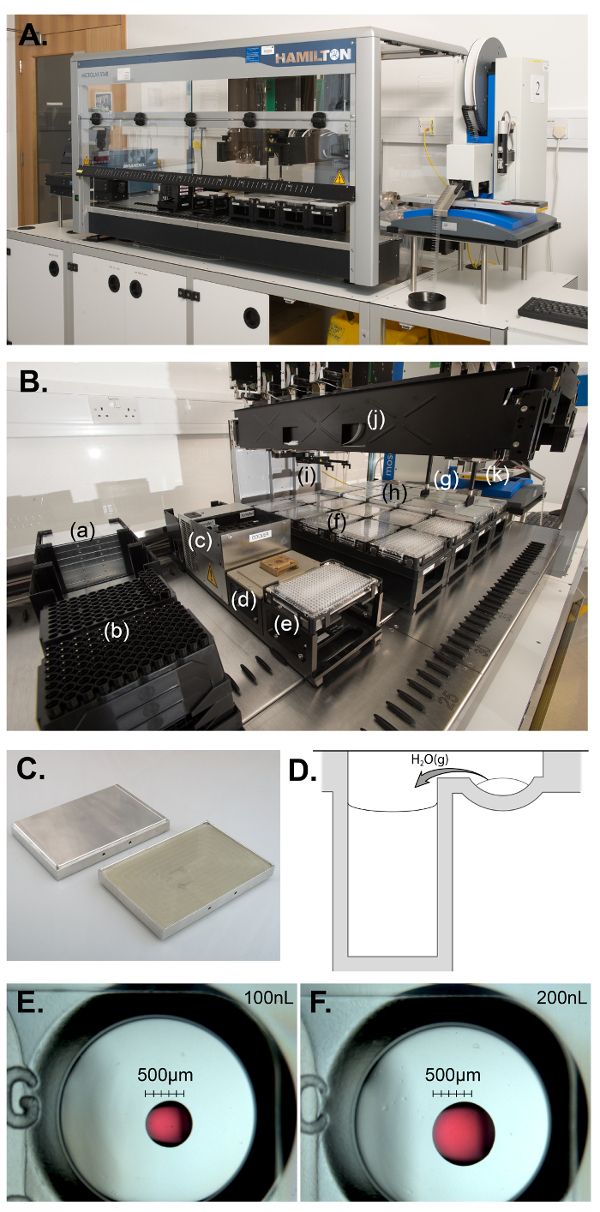

Figure 2**: Setting up droplets for vapor diffusion experiments. (A) Overview of the fully automated system 2.** (**B**) The main deck of the liquid handler in operation with (**a**) the storage carriers for stacks of SBS lids, (**b**) the stackable tips, (**c**) the cooling-carrier with a sample in microtube, (**d**) carrier for the 2 plate-moving adaptors, (**e**) the PCR plate for transferring protein to the nanoliter handling robot, (**f**) 9 sealed plates that have been placed back in their initial positions, (**g**) the optional gripper removing the SBS lid of the 10^th^ plate about to be transported onto the deck of the liquid handler, (**h**) the next 10 plates to be prepared with SBS lids on top, (**i**) the main gripper which is used to transport the PCR and crystallization plates, (**j**) the main automated arm integrating 12 pipetting probes (2 being used to operate as optional gripper) and the main gripper, and (**k**) the deck of the nanoliter dispenser. (**C**) In-house custom SBS lids made of aluminum. The lids integrate a rubber sheet to avoid evaporation of the conditions and two small holes to be accurately picked-up by the optional gripper. (**D**) Cross-section of a sealed crystallization well where the reservoir is filled with a condition and the upper-well contains a droplet composed from both the protein sample and the condition. Because the precipitant in the droplet is less concentrated than in the condition, the concentrations of all the components in the drop rise through water loss during the process of equilibration by vapor diffusion (very schematically represented by an arrow). (**E**) Light micrographs of 100 nL and (**F**) 200 nL droplets produced by the nanoliter dispenser with a test solution (20% v/v Polyethylene glycol 400, 0.001% w/v Safranin as red dye). The size and shape of the drops may vary according to chemicophysical properties of the condition. Please click here to view a larger version of this figure.

The main program starts with the optional gripper removing the SBS lid from the first crystallization plate. After the lid was transferred to the corresponding storage carrier, the crystallization plate is transported to the deck of the nanoliter dispenser. Then the pipetting arm transfers the amount of sample required for setting up droplets from the normally chilled microtube to the first column of the PCR plate. Subsequently, the main gripper moves the PCR plate onto the deck of the nanoliter dispenser that will prepare the droplets following the options selected by the user at the start (*e.g*. droplet size). For this, the protein sample is first dispensed into the upper-wells (using a single set of 8 microsyringes and multi-dispensing), then the crystallization conditions are dispensed onto the protein droplets (each row now requires 8 new microsyringes to avoid cross-contamination). Upon completion of the program to set up droplets, the main gripper transports the corresponding plate to the plate sealer, then places the PCR plate back in its original position (more protein will be dispensed in the next column of the PCR plate for the following plate). Finally, the sealed crystallization plate is moved back to its original position on the deck: Vapor diffusion experiments have already started in this plate (Figure 2D). This cycle is repeated according to the number of plates. When needed, the optional gripper removes an empty tip rack allowing the pipetting arm to access more tips. The program takes 2 hr and 20 min and 440 µL of sample to prepare single droplets in 20 plates using 100 nL sample + 100 nL condition (**Figure 2E and 2F**).

When using the nanoliter dispenser as stand alone robot for two additional plates (see Protocol, step 1.2.3), the entire set of initial screening plates available in the 10 °C incubator can be set up (22 LMB plates x 96 conditions = 2,112 conditions).

**Table 2** shows the requirements for the main program according to user selections on the system 2.

**Table:** 

**Plates**		**Drop size (nL)**	**Sample(s)**		**Tip requirements**	**Duration**
**Number**	**Type**		**Vol. 1 (µL) **	**Vol. 2 (µL) **	**50 µL tips **	**Microsyringes**	
10	96-well	100	240	0	80	1040	1 h 12 min
20	96-well	100	440	0	160	2080	2 h 20 min
10	96-well	100	240	240	160	2080	1 h 45 min
20	96-well	100	440	440	320	4160	3 h 05 min
10	48-well	1000	624	0	80	560	1 h 10 min
20	48-well	1000	1208	0	160	1120	2 h 16 min

**Table 2: Examples of the main program options available on the system 2 for setting up crystallization droplets.** The necessary amount of protein sample, tips and microsyringes vary according to the program. The volumes of sample ‘vol. 1’ (drop 1) and ‘vol. 2’ (drop 2) are calculated as follow: (8 tips x required volume per well of PCR plate + 4 µL lost volume) x number of crystallization plates + 40 µL dead volume in microtube. The required volume per well of PCR plate for 100 nL droplets in 96-well crystallization plate is 2 µL while 6.8 µL is required for 1,000 nL droplets in a 48-well plate (dead volume in wells of the PCR plate: 0.8 µL). An additional volume of sample is taken into consideration (‘lost volume’) due to evaporation from the microtube and other losses (e.g. sample sticking on tips). For example, for 20 MRC plates, 100 nL, 1 drop protocol, the volume of sample required is: (8 x 2 + 4) x 20 + 40 = 440 µL (*i.e.*, the equivalent of 22 µL per plate). Eight disposable 50 µL tips are required for each plate and each sample. For example, for 10 plates, 2-drop protocol, the number of tips required is 8 x 10 x 2 = 160 tips. The number of microsyringes is calculated as follow: (8 + number of conditions per plate) x number of samples x number of plates. For example, for 10 x 48-well plates, the number of liquid handler tips required is: (8 + 48) x 1 x 10 = 560 tips.

**3. Formulation, preparation and handling of the 4-corner solutions**

Both formulations of the 4 corner solutions (‘A, B, C and D’) and the corresponding optimization screen (Figure 3A) are automatically generated by an Excel spreadsheet. There are different spreadsheets for different numbers of conditions — essentially 24, 48, and 96 conditions — and also spreadsheets to prepare two different optimization screens in a single plate. One typically prepares a set of 4 x 10 mL corner solutions in test tubes from which 2–3 optimization screens can be prepared, depending on the number and volume of conditions required. The solutions are poured into troughs from which they are aspirated by a syringe-based liquid handler and later dispensed directly into plates (Figure 3B). The two linear gradients of concentrations result from mixing A, B, C, and D at systematically varying ratios (Figure 3C).

**Figure:**
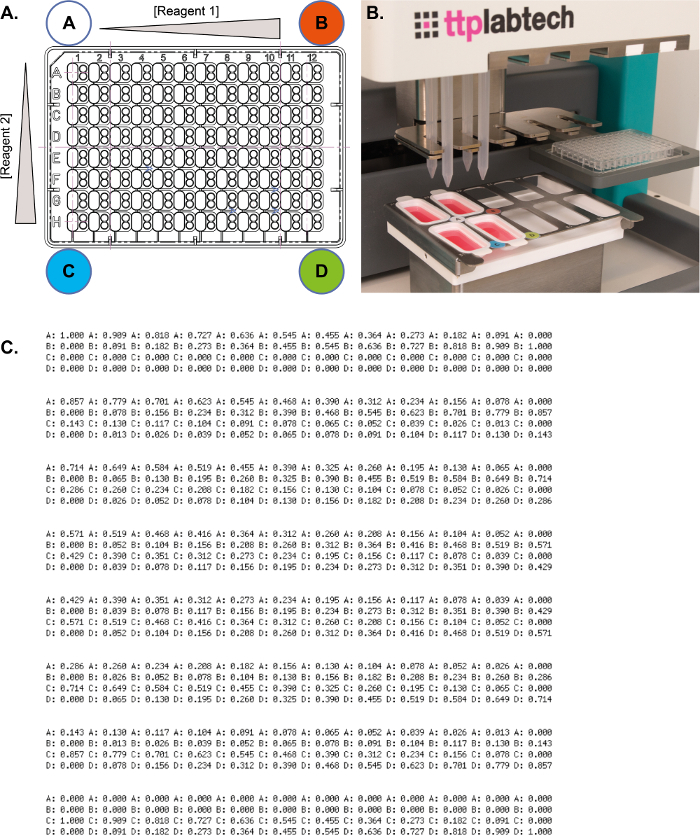

**Figure 3: The 4-corner method for preparing optimization screens.** (**A**) Representation of the two gradients of concentrations across a 96-well plate layout. (**B**) The syringe-based liquid handler ready to prepare a screen with 4 syringes inserted into the head of the robot. A crystallization plate sits in the motorized SBS carrier and the 4 starting solutions (A, B, C, and D) have already been dispensed into their respective troughs (the solutions have been colored red for demonstration purposes only). (**C**) Ratios of the solutions A, B, C, and D across 96 reservoirs. Please click here to view a larger version of this figure.

**Table 3** shows the requirements for the programs available to users on the syringe-based liquid-handler.

**Table:** 

**Plate type**	**No. of conditions **	**Vol. in plate (reservoirs, µL) **	**Vol. in trough (mL)**	**Duration**
96-well	48	80	1.6	2 min 5 sec
96-well	96	80	2.5	3 min 50 sec
96-well	2 x 48	80	1.6	2 min 50 sec
48-well	24	200	1.8	2 min 25 sec
48-well	48	200	3	4 min 20 sec

**Table 3: Programs available on the syringe-based liquid handler for preparing optimization screens according to the 4-corner method.** The 96-well plate can be used to prepare 96- or 48-condition optimization screen (when two 48-condition screens are prepared simultaneously, the robot must be equipped with 8 syringes and 8 troughs). Other programs enable the use of 48-well plates. The listed volumes in troughs include the required dead volume (0.5 mL).

**4. Additive screening**

Figure 4 shows the steps to perform an additive screening starting either with 96 additive solutions already in the reservoirs of the crystallization plate (protocol 1) or in the wells of a low-profile cell culture plate used as a re-usable additive screen (protocol 2). **Table 4** lists the programs available on the ‎nanoliter dispenser according to the two types of protocols.

**Figure:**
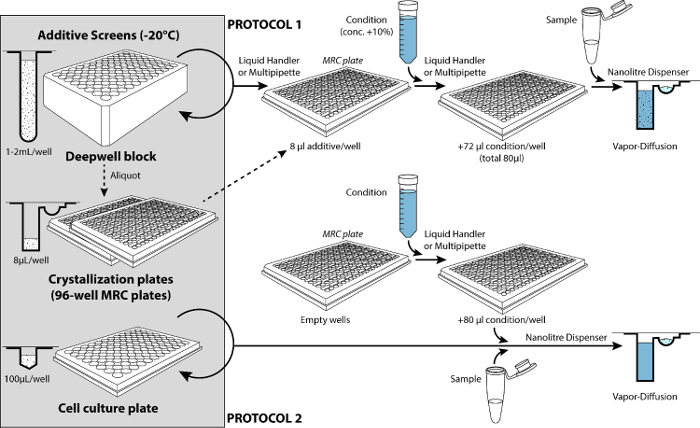

**Figure 4: The two types of protocols for additive screening.** The 96-additive screens are stored at -20 °C. Following protocol 1, the additive screen is added initially to the reservoirs of the crystallization plates (and ideally stocked this way). A liquid handler or multipipette is used to dispense the condition into the reservoirs containing additive solutions (The volume of additive screen represents 10% of the final volume in reservoirs: 8 µL for 80 µL final volume). After mixing the conditions on a microplate mixer (not shown), the microsyringe-based nanoliter dispenser is used to set up the droplets (**Table 4**). Following protocol 2, the additive screen is added only later while setting up the crystallization droplets. This time the nanoliter dispenser is employed to prepare droplets with an additional component on its deck (the re-usable cell culture plate containing the additive screen). Please click here to view a larger version of this figure.

**Table:** 

**Protocol**	**Drop size (nL)**		**Strip type**	**Sample vol. (µL)**		**No. of microsyringes**	**Duration**
**Type**	**Source of additives**	**Protein**	**Condition**	**Additive**		**In each well (strip)**	**Total**		
1	96-well crystallization plate	100	100	0	2 µL	2	16	104	2 min
1	96-well crystallization plate	200	200	0	5 µL	3.8	31	104	2min 10 sec
2	96-well cell culture plate	200	200	100	5 µL	3.8	31	200	3min 45 sec
2	96-well cell culture plate	500	500	100	5 µL	7.4	60	200	4min 15 sec

**Table 4: Programs available on the microsyringe-based nanoliter dispenser for additive screening.** The required volume of sample in each well of the 8-well strip is calculated as follows: dead volume + 12 x drop size. There are 2 types of strips (2 µL and 5 µL). The dead volumes are 0.8 µL for the 2 µL wells (which can actually contain 3.2 µL max.) and 1.4 µL for the 5 µL wells (7.5 µL max.). The total volume of protein required is: 8 x volume in well of the strip (round-up value). The dead volume of the V-shaped wells of the 96-well cell culture plate is 2.5 µL. The required number of microsyringes varies according to the selected program (8 + 96 = 104; or 8 + 2 x 96 = 200).

Because of the dilution of the condition with the additive during protocol 1, the condition needs to be prepared at a proportionally higher concentration than initially. The increase in final concentrations is most easily achieved by reducing the final addition of water to the calculated final volume. After this, one simply proceeds with the normal set up of droplets that mix the condition and sample (*e.g.*, 100 nL protein + 100 nL condition already mixed with additives). Protocol 2 (*e.g.*, 200 nL protein + 200 nL condition + 100 nL additive) facilitates the screening at different concentrations of additives by simply varying the volume of additive screen added. Protocol 2 implies more or less dilution of the droplets (which may alter crystallization).

The liquid handler from system 2 can be used to aspirate enough condition in 12 tips and dispense 8 aliquots into the reservoirs of a 96-well plate (see **Protocol**, step 2.2.2), although this step can of course be done manually with a multichannel pipette (Figure 4). Several plates can be filled at a time with the same condition when using the liquid handler (to test different additive screens later). When preparing 2 plates, fill the reagent container with at least 23 mL. When preparing 3 plates, fill the reagent container with at least 31 mL.

**5. Crystallization plates and associated devices**

The design of both MRC sitting-drop vapor diffusion crystallization plates (96-well 2-drop and 48-well 1-drop, Figure 5A and **Figure 5B**) provides characteristics that enable reliable and efficient automated crystallization experiments, with notably the spherical shape of the upper-wells and a slight V-shape of the reservoirs which facilitate accurate dispensing and also centrifugation when centering of the droplets is required. In addition, the upper-wells have a lens effect for optimal illumination under a stereomicroscope (The polymer is UV transmissible for the detection of UV-absorbing or fluorescent crystals^23^).

A custom seal (Figure 5C) enables setting up hanging drop crystallization experiments within both types of plates using a nanoliter dispenser (protocols not shown). Finally, both MRC plates have the same outer dimensions and rim. The rim fits into the grooves of our custom-built plate holder (Figure 5D), which is used for manually placing/removing sealing tape without splashing liquids.

**Figure:**
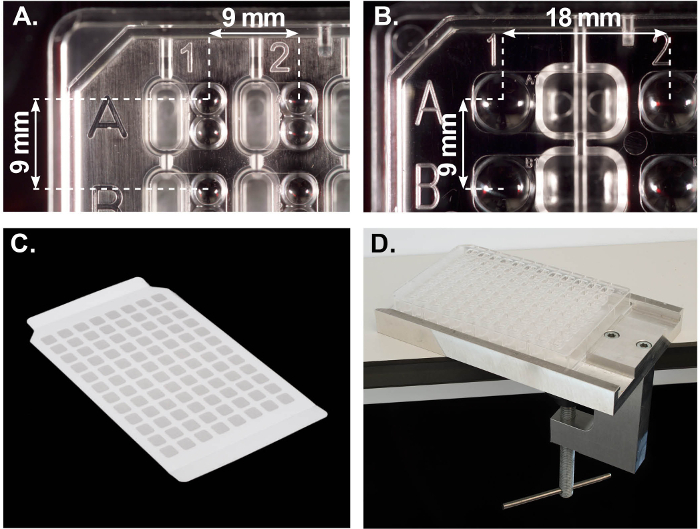

**Figure 5: The MRC crystallization plates and associated devices developed at the LMB.** (**A**) A1-corner of the 96-well plate. The reservoir (oblong) is on the left-hand side of the crystallization well, the two spherical upper-wells on the right. Dimensions are in millimeters. (**B**) A1-corner of the 48-well plate. The reservoir is on the right-hand side of the well (1 large upper-well only). (**C**) MRC hanging drop seal. (**D**) In-house custom plate holder. Please click here to view a larger version of this figure.

**6. Protein crystals**

Figure 6 shows examples of useful crystals obtained with our automated protocols.

**Figure:**
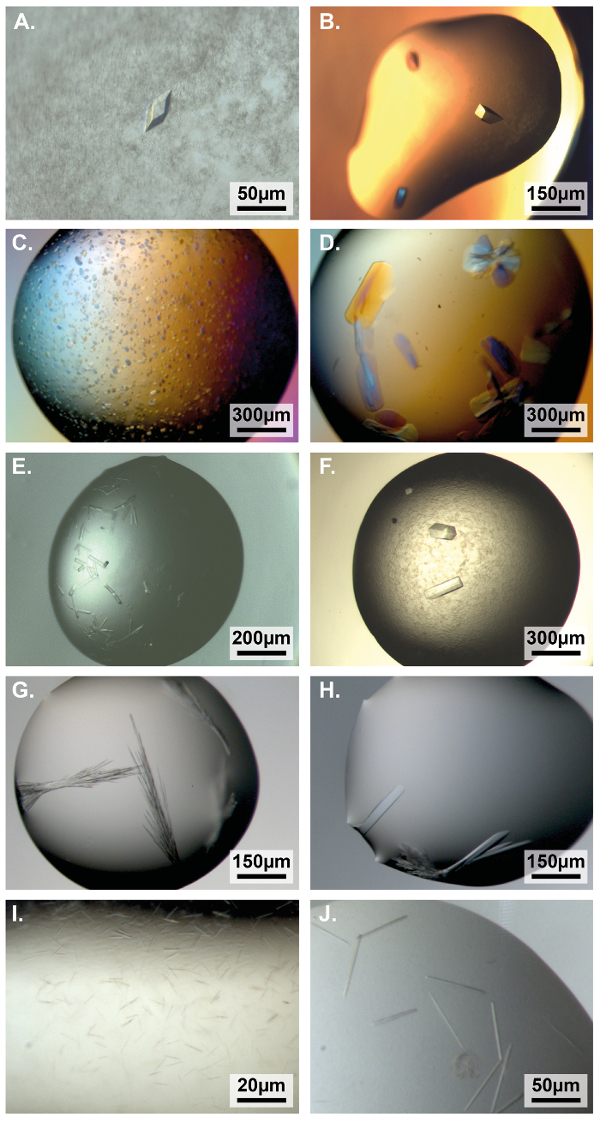

**Figure 6: Light micrographs of droplets containing crystals obtained following the automated protocols.** (**A**, **B**) Crystals from initial screen of OmpF and MreB in plates prepared with the 2 fully automated systems (see **Protocol** sections 1.1 and 1.2, conditions: LMB07 well A4 and LMB20 well D12, respectively, size of droplets is 100 nL protein + 100 nL condition, work of Andrzej Szewczak, LMB)^24^. (**C**, **D**) Bar domain crystals before and after optimization of initial conditions with the 4-corner method (step 2.1, LMB02 B6, 1,000 nL + 1,000 nL, unpublished work of Leonardo Almeida-Souza, LMB). (**E**, **F**) Heavy chain of human dynein 1 N-terminal domain crystals before and after optimization of the initial conditions with the 4-corner method (LMB20 E6, 200 nL + 200 nL, then 500 nL + 500 nL, unpublished work of Edgar Morales-Ríos, LMB). (**G**, **H**) Complement factor D crystals before and after optimization of the condition with additive screening (step 2.2, initial condition from in-house custom screen, 200 nL + 200 nL, additive D6 from the 96-condition additive screen, unpublished work of Matthias Bauer, LMB). (**I**, **J**) Viral envelope glycoprotein^25^ crystals before and after optimization of the condition with additive screening (step 2.3, LMB20 A2, 150 nL + 150 nL, then 200 nL + 200nL + 100 nL additive E5 from the 96-condition additive screen, unpublished work of Yorgo Modis, University of Cambridge, UK). Please click here to view a larger version of this figure.

## Discussion

**1- Preparation and use of initial screens stored in plates**

Screening kits should be mixed before being dispensed into plates because light precipitation or phase separation occurs in some tubes during storage. When a screen is composed of two kits (2 x 48 tubes), the first tube of the second kit is placed in in location E1 of the cooling carrier. When a screen is composed of 4 kits (4 x 24 tubes), the first tube of the second kit is placed in in location C1, the first tube of the third kit is placed in location E1 and the first tube of the fourth kit is placed in location G1. While inserting the tubes in their cooling carrier, lids are placed on a tray following the standard 96-well landscape layout. Since well numbers are indicated on top of the lids by the manufacturers, this enables cross-checking if all tubes have been placed in the right order. This also helps to replace the correct lids on the tubes when filling a reduced number of plates.

We store the pre-filled plates at 10 °C, a compromise to avoid freezing and storage at 4 °C that may cause deterioration of conditions and issues with sealing. Plates are stored for up to several months with normally no noticeable condensation on the inner face of the seal. This is less true for LMB05, LMB06, LMB09 and LMB10 plates as these contain conditions with relatively high concentrations of volatile reagents (**Table 1**). Small amount of condensation on the inner side of the seal reduces sealing efficiency and can cause cross-contamination between wells while unsealing the plates. To help with preventing condensation during initial cooling, plates can be first transferred from the carousel into an insulated picnic cooler which is stored in a 4 °C cold room overnight. The very slow cooling minimizes the development of temperature gradients within the sealed wells and hence reduces condensation overall^15^. In addition, once the plates are stored in the 10 °C incubator, an in-house custom SBS polystyrene lid is placed on the plate at the top of each stack (not shown).

The entire set of our pre-filled plates can be used as a large initial screen against a novel, water-soluble, protein sample. Alternatively, fewer plates may be selected to match specific requirements. For example, LMB15 and LMB19 are screens formulated specifically for membrane protein samples^26^,^27^, or LMB20 is a screen formulated with heavy-atoms to facilitate the experimental phasing of diffraction data^28^ (see also: Formulation of the MORPHEUS protein crystallization screens).

**2. Setting up crystallization droplets**

When using the system 2, screening kits with significant amounts of volatile reagents should be processed first. This avoids condensation forming on the rubber of the SBS lids, which could affect lid handling and plate sealing. An SBS lid has a bit of clearance when on the top of a plate, which is why they need to be aligned initially (see **Protocol**, step 1.2.6). The protein dead volumes in the wells of the PCR plate are relatively generous (0.8 µL, see legend of **Table 2**). Note that equally generous dead volumes are employed when using the nanoliter dispenser individually with protein in 8-well strips (**Table 4**). Smaller dead volumes may work, however some samples adhere to the tips, calibration of a robot may become slightly inaccurate, the room may be warmer than usual, *etc*. All lead to sample losses covered by the generous dead volumes in order to consolidate the approach.

Recent developments enabled further miniaturization of experiments and hence the volume of sample required for screening crystallization conditions can be significantly reduced by integrating the corresponding technology^29^,^30^. However, some aspects of further miniaturization need careful consideration, such as the evaporation of droplets^31^ and the manipulation of microcrystals^32^.

Finally, centrifugation of the plate (2,000 rpm, 1 min) could be integrated as a routine final step when setting up crystallization droplets (in spherical upper-wells). A more consistent size and shape of droplets resulting from centrifugation may reduce reproducibility issues^33^,^34^. Surely, centered drops will ease the later assessment of experiments using a microscope as the required focal length will be similar across the entire plate.

**3. Advantages of the 4-corner method**

The most significant advantage of the 4-corner method is its simplicity, which minimizes errors and facilitates straightforward automated protocols. For example, the 4 corner solutions will always be placed on the deck of a liquid handler following the same layout. Also, all programs are based on fixed ratios between the solutions (**Figure 3C**). Manual preparation of the 4 corner solutions is preferred to automated handling of solutions at high concentrations which can be highly viscous. Relatively fast and accurate aspiration/dispensing is then possible on most types of liquid handlers with minimum requirements for optimization of liquid classes. Nevertheless, some corner solutions may still be too viscous for a robot operating with a liquid-system to operate efficiently. This is why we opted for a liquid handler operating with positive displacement (**Figure 3B**).

In addition to the 2 linear gradients of concentrations, a third component (*i.e.*, a set of buffers/additives) can be tested at a constant concentration in a convenient way. For this, a relatively large volume of a core set of corner solutions at a suitably higher concentration, excluding the component to be varied, is prepared first. Then, stock solutions including this component is added to adjust the final concentrations. For example, 50 mL of a set of 4 corner solutions are prepared at 10 % higher concentrations than initially. This core set is then split into 5 smaller subsets of 4. Finally, 10% in volume of different buffer-pH solutions is added to each subset.

**4. Formats and types of additive screens**

The screens are normally stored at -20 °C (Figure 4) since they are not used regularly and contain volatile/unstable compounds. The use of a frozen additive screen stored in a deep well block (1 mL in wells) must be planned early because it will take 12-24 hr for all the additive solutions to thaw completely at room temperature. Also, a multitude of users share the same additive screen, potentially causing problems with cross-contamination. Finally, the height of deep well blocks makes them unsuitable for most nanoliter dispensers. As a convenient solution to circumvent these issues, the screen should be transferred from the deep well block to low-profile plates (Figure 4).

Historically, additive screens which include a broad variety of single reagents (with single concentrations) have been very popular^35^,^36^. However, other types of additive screens have been developed that integrate mixes of additives^37^ or a reduced number of single additives found at different concentrations^38^. Finally, a complementary approach is to investigate the effect of additives on the samples prior to crystallization^39^,^40^.

**5. More considerations**

**Good practice:** Most screens contain harmful or even toxic substances and hence adequate personal protection must be employed during the protocols. Equally, moving parts of the robots may lead to injuries, especially when trying to manually interfere while a program is running (although most of the robots have emergency stop button/system). Because of the technical complexities involved, regular checking of robots, screens and programs with previously characterized test samples are important for sustained high levels of reproducibility.

**Throughput:** As an indication, between 4,000 to 8,000 LMB plates are produced yearly with the system 1 (and subsequently employed by users for initial screening). It is not adapted to stock a large amount of pre-filled plates at 10 °C when the expected turnover is much lower, as after 4-5 months, some conditions will start to deteriorate and evaporate. Different approaches to automation protocols have been implemented for small- to medium-size laboratories^41^.

**Storing and assessing experiments:** After preparing the droplets, plates are stored on low-vibration shelves in a room at 4 or 18 °C with tightly controlled temperature (+/- 0.5 °C maximum deviation). Experiments are assessed using cold light source microscopes. Various automated imaging systems are commercially available, however one should carefully consider all aspects: Will the speed required to scan a plate be sufficient for high throughput? Will objects other than crystals interfere with autofocus? Will the resulting quality of images be sufficient to spot very small crystals (especially around the edge of the droplets)?^42^,^43^,^44^

**Comparison of crystallization conditions:** After careful investigations about the nature of the initially-obtained crystals, one can analyze trends and similarities across conditions using the LMB screen database or the C6 Web Tool^45^.

## Disclosures

We hereby state a conflicting commercial interest since LifeArc commercializes the following items: the 96- and 48-well MRC plates, the MRC hanging drop seal, the MORPHEUS, Pi, ANGSTROM and LMB crystallization screens.
